# Supplementary material for: Force-activated zyxin assemblies coordinate actin nucleation and crosslinking to orchestrate stress fiber repair
Source: bioRxiv. 2024 May 18:2024.05.17.594765. Preprint. [Version 1] doi: 10.1101/2024.05.17.594765 (PMC11118565; doi:10.1101/2024.05.17.594765)
Supplement: 8 [file NIHPP2024.05.17.594765V1-supplement-1.pdf]

bioRxiv preprint doi: https://doi.org/10.1101/2024.05.17.594765; this version posted May 18, 2024. The copyright holder for this preprint (which was not certified by peer review) is the author/funder, who has granted bioRxiv a license to display the preprint in perpetuity. It is made available under aCC-BY 4.0 International license.

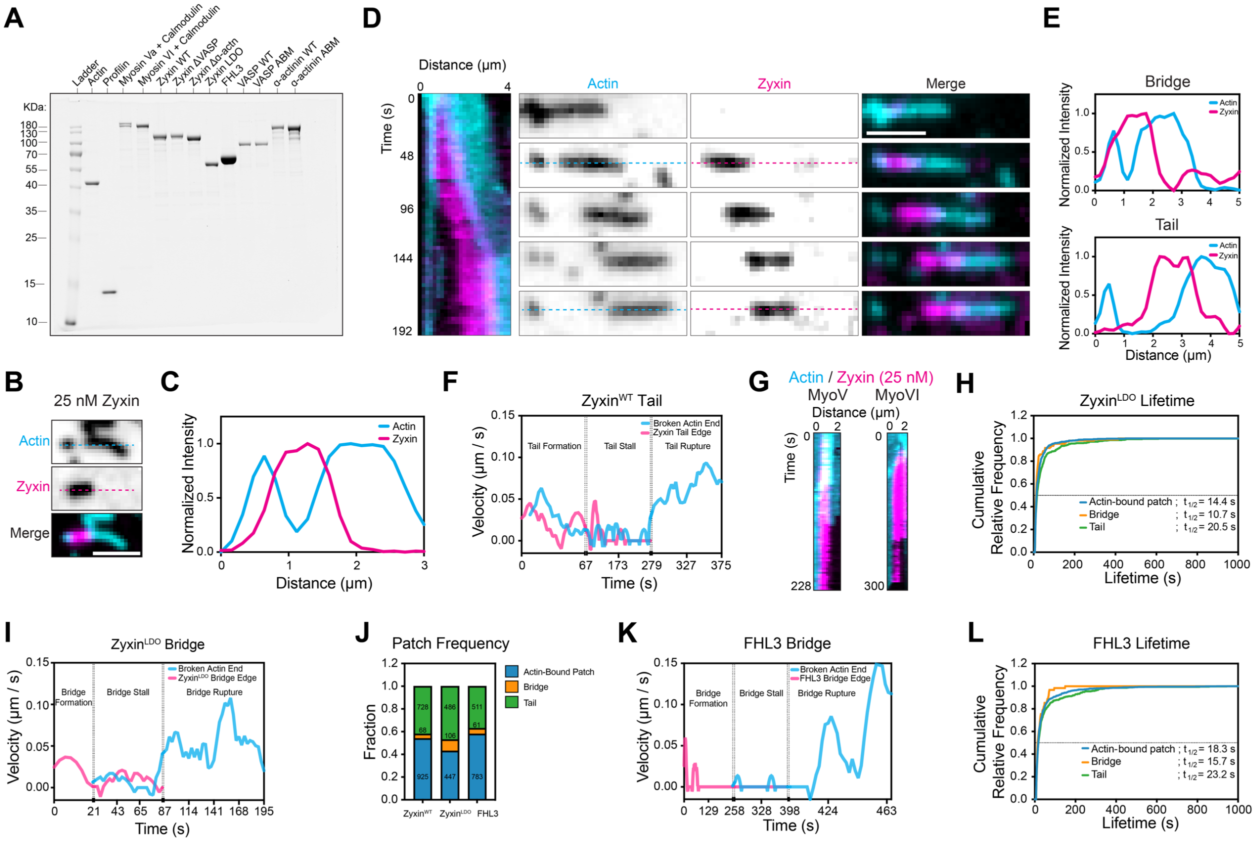

Figure S1. Zyxin bridges and tails feature similar architectures and dynamics. Related to Figure 1. **A)** Coomassie-stained SDS-PAGE of purified proteins. **B)** TIRF snapshot of a zyxin bridge formed in the presence of 100% ATTO 488-labeled actin filaments (cyan) and 25 nM zyxin-Halo:JF646 (magenta). Scale bar, 2 µm. **C)** Normalized fluorescence intensity along dashed lines shown in B. **D)** Kymograph (left) and montage (right) of a zyxin bridge forming in the presence of 250 nM zyxin-Halo:JF646, then converting into a tail. Time labels correspond to both montage snapshots and kymograph. Scale bar, 2 µm. **E)** Normalized fluorescence intensity along dashed lines shown in D (bridge, 48 s; tail, 192 s). **F)** Instantaneous velocity over time of the interface between a zyxin tail and a broken F-actin fragment. **G)** Kymographs of zyxin bridges converting into tails in the presence of myosin Va alone (left) or myosin VI alone (right). Zyxin-Halo:JF646, 25 nm. **H)** Cumulative relative frequency of zyxin LDO actin-bound patch, bridge, and tail lifetimes. Dotted line = 0.5. **I)** Instantaneous velocity over time of the interface between a zyxin LDO bridge and a broken F-actin fragment. **J)** Patch category frequency across LIM proteins. Number of each structure observed in each category are indicated; N ≥ 2 biological replicates. **K)** Instantaneous velocity over time of the interface between a FHL3 bridge and a broken F-actin fragment. **L)** Cumulative relative frequency of FHL3 actin-bound patch, bridge, and tail lifetimes. Dotted line = 0.5.

bioRxiv preprint doi: https://doi.org/10.1101/2024.05.17.594765; this version posted May 18, 2024. The copyright holder for this preprint (which was not certified by peer review) is the author/funder, who has granted bioRxiv a license to display the preprint in perpetuity. It is made available under aCC-BY 4.0 International license.

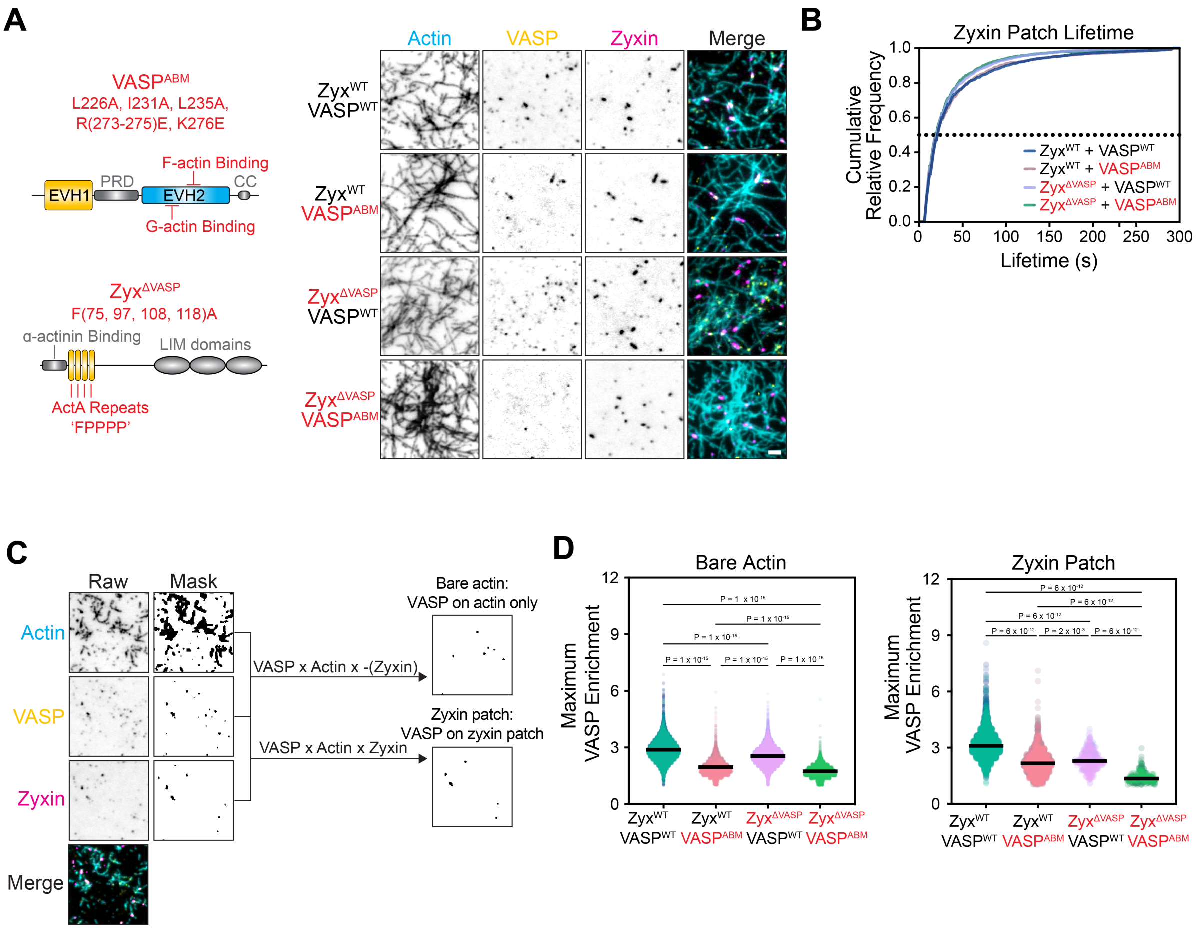

Figure S2. Additional analysis of VASP enrichment on zyxin patches. Related to Figure 2.
A) Left: Schematic of zyxin$^{\Delta VASP}$ and VASP^{ABM} mutant constructs. Right: TIRF snapshots of force reconstitution assays featuring the indicated constructs, performed with 30% ATTO 488-labeled actin filaments (cyan), 50 nM VASP-Halo:JF549 (yellow), and 25 nM zyxin-Halo:JF646 (magenta). Scale bar, 2 µm. **B)** Cumulative relative frequency of zyxin patch lifetimes in the presence of the indicated constructs. Dotted line = 0.5. **C)** Workflow for quantifying VASP on bare F-actin vs. actin-bound zyxin patches.
D) Maximum VASP enrichment on bare F-actin (left) or zyxin patches (right) in the presence of the indicated constructs. Bars represent means; $367 \leq n \leq 4479$ from N = 2 biological replicates (represented by shades). Conditions were compared by Turkey's multiple comparisons test after ordinary one-way ANOVA.

bioRxiv preprint doi: https://doi.org/10.1101/2024.05.17.594765; this version posted May 18, 2024. The copyright holder for this preprint (which was not certified by peer review) is the author/funder, who has granted bioRxiv a license to display the preprint in perpetuity. It is made available under aCC-BY 4.0 International license.

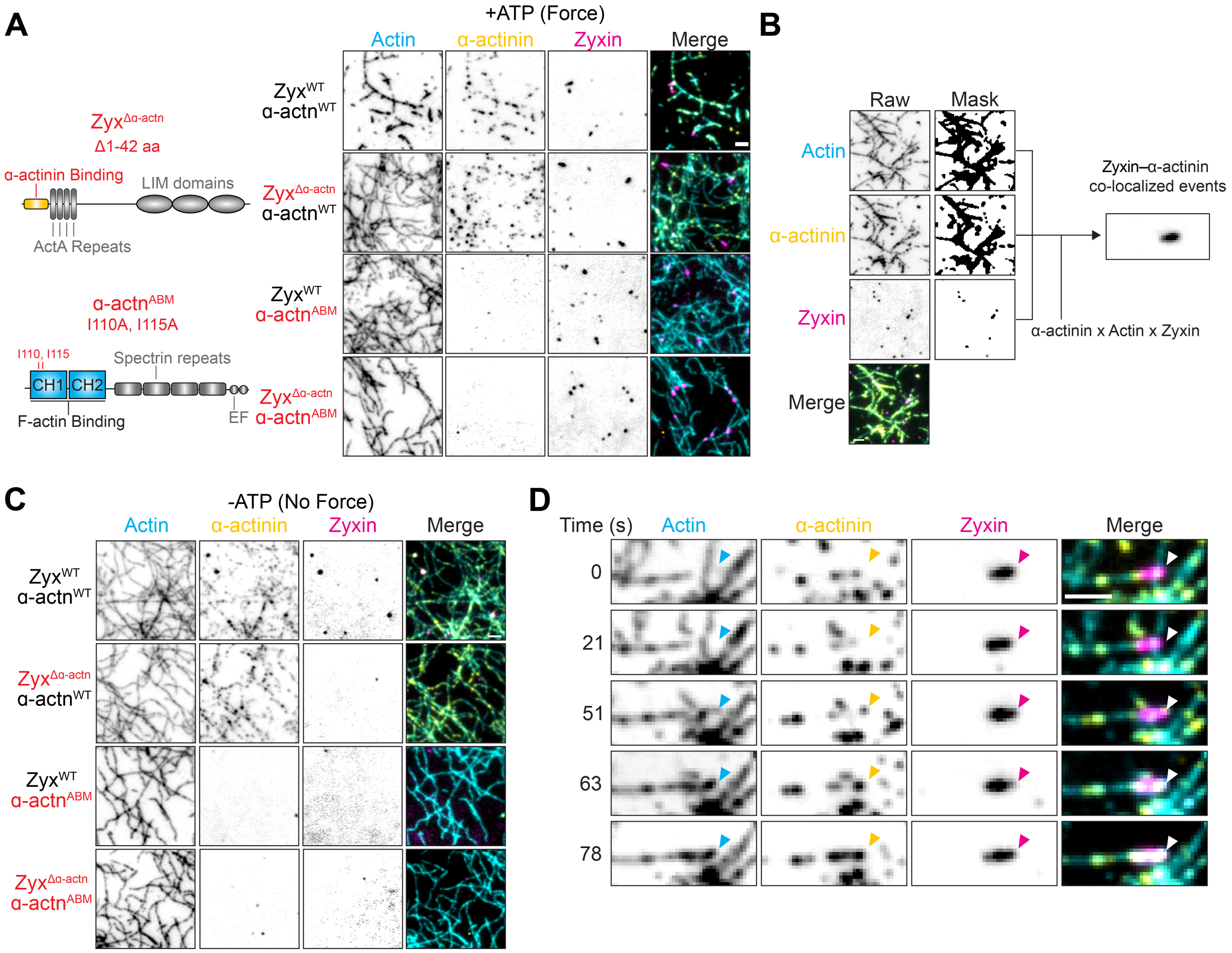

Figure S3. F-actin bound α-actinin weakly engages zyxin patches. Related to Figure 3.
A) Left: Schematic of zyxin$^{\Delta\alpha\text{-actinin}}$ and α-actinin^{ABM} mutant constructs. Right: TIRF snapshots of force reconstitution assays performed with 30% ATTO 488-labeled actin filaments (cyan), 50 nM α-actinin-Halo:JF549 (yellow), and 25 nM zyxin-Halo:JF646 (magenta) in the presence of the indicated constructs and immobilized myosin forces (+ATP). Scale bar, 2 μm. **B)** Workflow for quantifying zyxin–α-actinin colocalization events. **C)** TIRF snapshots of reconstitution assays performed as in A, but in the absence of force generation (-ATP). Scale bar, 2 μm. **D)** Montage of multiple actin filaments (cyan) bundling along a zyxin patch (magenta) through α-actinin clusters (yellow). Patch position is indicated with arrowheads. The assay was performed with 30% ATTO 488-labeled actin filaments, 50 nM α-actinin-Halo:JF549, and 25 nM zyxin-Halo:JF646. Scale bar, 2 μm.

bioRxiv preprint doi: https://doi.org/10.1101/2024.05.17.594765; this version posted May 18, 2024. The copyright holder for this preprint (which was not certified by peer review) is the author/funder, who has granted bioRxiv a license to display the preprint in perpetuity. It is made available under aCC-BY 4.0 International license.

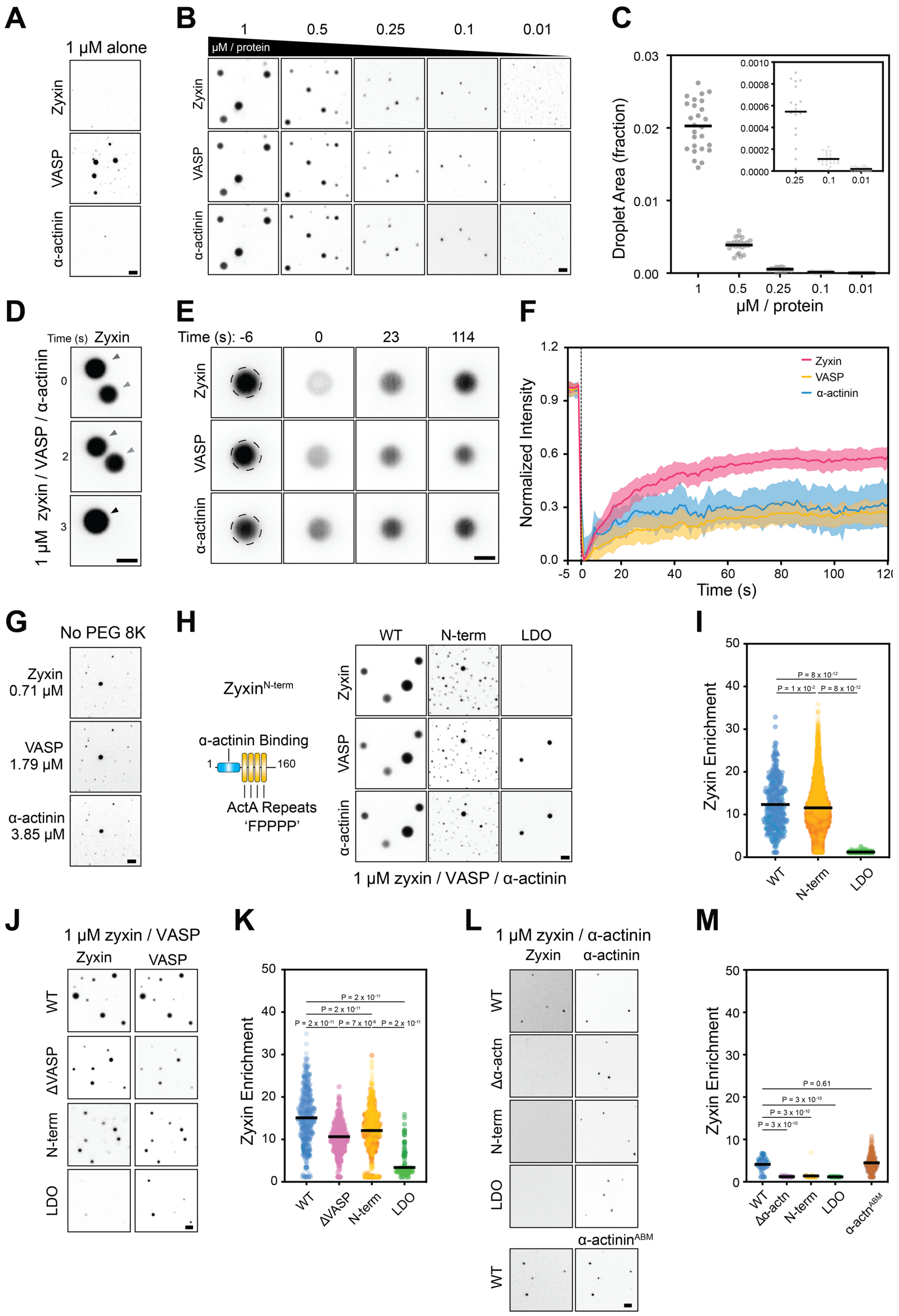

Figure S4. Zyxin, VASP, and α-actinin form droplets through multivalent interactions. Related to Figure 5.

A) iSIM snapshots of 1 μM zyxin-Halo:JF646 (top), 1 μM VASP-Halo:JF549 (middle), and 1 μM α-actinin-Halo:AF488 (bottom), individually in the presence of 3% PEG 8K. Scale bar, 2 μm. **B)** iSIM snapshots of tripartite droplets across varying concentrations of zyxin-Halo:JF646, VASP-Halo:JF549, and α-actinin-Halo:AF488 in the presence of 3% PEG 8K. Scale bar, 2 μm. **C)** Quantification of droplet size distribution (as fraction of field of view) from experiments presented in B. n = 12 fields from N = 2 biological replicates. **D)** Montage of two droplets undergoing fusion. Tripartite droplets were assembled from 1 μM zyxin-Halo:JF646, 1 μM VASP-Halo:JF549, and 1 μM α-actinin-Halo:AF488 in the presence of 3% PEG 8K. Scale bar, 2 μm. **E)** Montage of fluorescence recovery after photobleaching (FRAP) of a tripartite droplet prepared as in D. Dashed circle represents the region illuminated at time = 0 s. Scale bar, 2 μm.

bioRxiv preprint doi: https://doi.org/10.1101/2024.05.17.594765; this version posted May 18, 2024. The copyright holder for this preprint (which was not certified by peer review) is the author/funder, who has granted bioRxiv a license to display the preprint in perpetuity. It is made available under aCC-BY 4.0 International license.

F) Quantification of experiments presented in E. Dashed line represents time = 0 s. Data are presented as mean ± SD; n = 14 droplets bleached from N = 2 biological replicates. **G)** iSIM snapshot of tripartite droplets formed with indicated physiological concentrations of zyxin-Halo:JF646, VASP-Halo:JF549, and α-actinin-Halo:AF488 in the absence of PEG 8K. Scale bar, 2 μm. **H)** Left: Schematic of zyxin^{Nterm} construct. Right: iSIM snapshots of tripartite droplets formed with indicated zyxin constructs in the presence of 3% PEG 8K. Samples were prepared with 1 μM zyxin-Halo:JF646, 1 μM VASP-Halo:JF549, and 1 μM α-actinin-Halo:AF488. Scale bar, 2 μm. **I)** Quantification of zyxin enrichment in droplets from experiments presented in H. Bars represent means; 338 ≤ n ≤ 5103 from N = 2 biological replicates (represented by shades). **J)** iSIM snapshots of bipartite zyxin and VASP droplets formed with indicated zyxin constructs in the presence of 3% PEG 8K. Samples were prepared with 1 μM zyxin-Halo:JF646 and 1 μM VASP-Halo:JF549. Scale bar, 2 μm. **K)** Quantification of zyxin enrichment in droplets from experiments presented in J. Bars represent means; 203 ≤ n ≤ 651 from N = 2 biological replicates (represented by shades). **L)** iSIM snapshots of bipartite zyxin and α-actinin droplets formed with indicated zyxin and α-actinin constructs in the presence of 3% PEG 8K. Samples were prepared with 1 μM zyxin-Halo:JF646 and 1 μM α-actinin-Halo:AF488. Scale bar, 2 μm. **M)** Quantification of zyxin enrichment in droplets from experiments presented in L. Bars represent means; 34 ≤ n ≤ 359 from N = 2 biological replicates (represented by shades). All statistical comparisons were performed with Turkey's multiple comparisons test after one-way ordinary ANOVA.

bioRxiv preprint doi: https://doi.org/10.1101/2024.05.17.594765; this version posted May 18, 2024. The copyright holder for this preprint (which was not certified by peer review) is the author/funder, who has granted bioRxiv a license to display the preprint in perpetuity. It is made available under aCC-BY 4.0 International license.

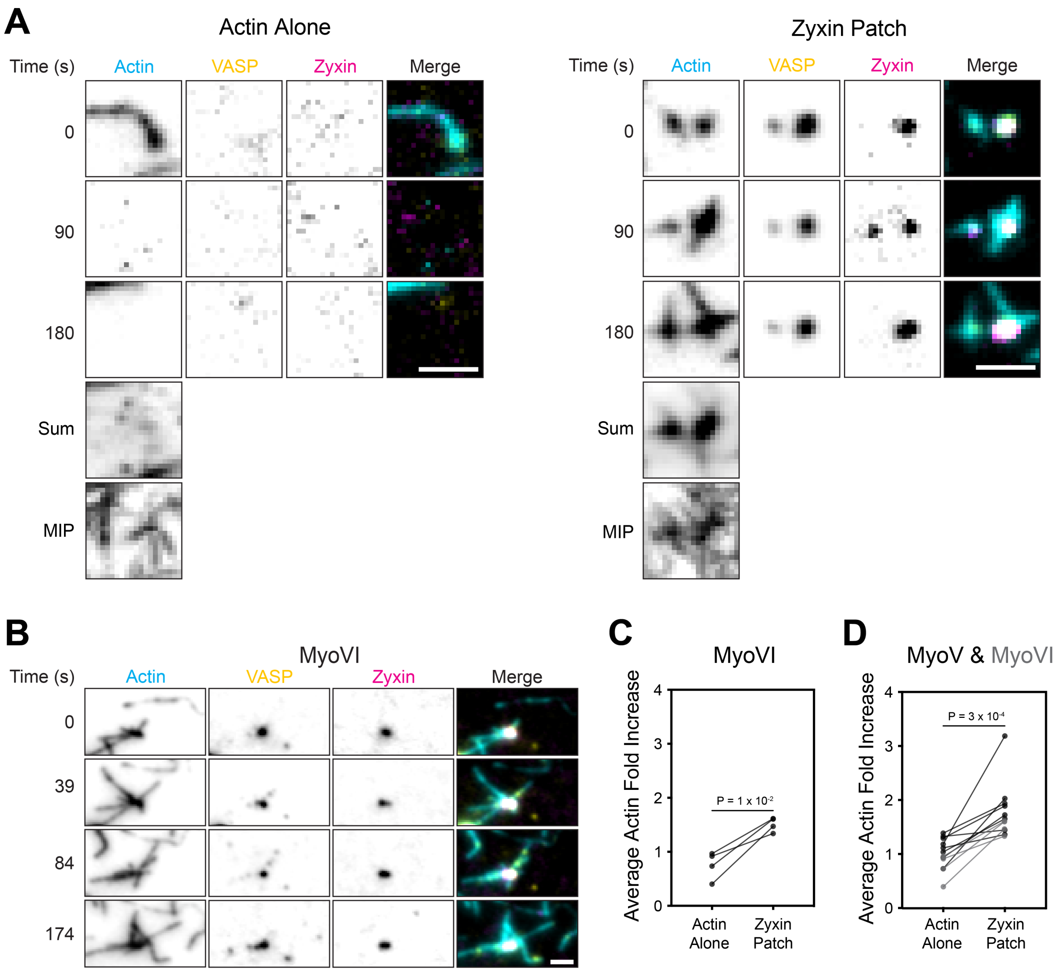

Figure S5. Zyxin patches promote VASP-mediated actin nucleation and polymerization. Related to Figure 4.
A) Montages of undetectable actin filament (cyan, 30% ATTO 488-labeled) nucleation and polymerization at a bare F-actin region (left) versus at a VASP-enriched (yellow) zyxin patch (magenta, right) in the presence of immobilized myosin VI forces (+ATP). Assay was performed with 0.5 µM 30% ATTO-488 G-actin, 2 µM profilin, 50 nM VASP-Halo:JF549, and 25 nM zyxin-Halo:JF646. Scale bar, 2 µm. **B)** Montage of F-actin nucleation and polymerization-coupled extrusion from a zyxin patch formed in the presence of myosin VI (+ATP). Assay conditions were otherwise identical to Figure 4B. **C)** Pairwise comparison of the average actin intensity fold increase at VASP-enriched zyxin patches versus bare F-actin regions in the presence of myosin VI forces (+ATP). Assays were performed as in A. n = 4 trials (imaging 1-5 field of view per trial) from N = 2 biological replicates, compared by paired t test. **D)** Pooled paired comparisons of average actin fold increase at VASP-enriched zyxin patches vs. bare F-actin across trials, in the presence of either immobilized myosin V (black) or myosin VI (grey) individually. n = 12 trials from N = 2 biological replicates, compared by paired t test.

bioRxiv preprint doi: https://doi.org/10.1101/2024.05.17.594765; this version posted May 18, 2024. The copyright holder for this preprint (which was not certified by peer review) is the author/funder, who has granted bioRxiv a license to display the preprint in perpetuity. It is made available under aCC-BY 4.0 International license.

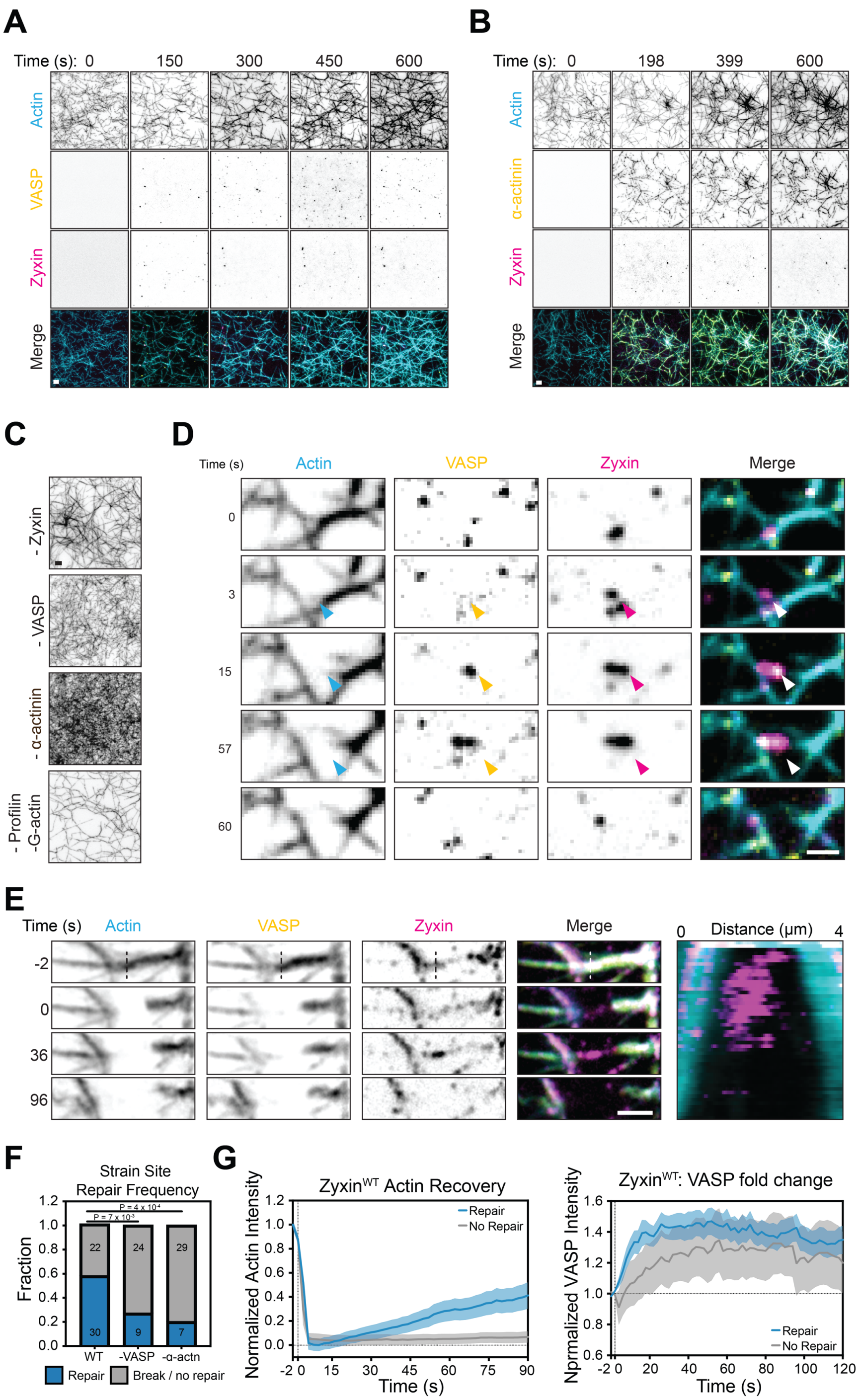

Figure S6. Further characterization of reconstituted contractile bundles and F-actin repair. Related to Figure 5.
A) Montage of bundle reconstitution assay featuring labeled VASP and unlabeled α-actinin. Assay was prepared with 30% ATTO 488-labeled F-actin (cyan), 25 nM zyxin-Halo:JF646 (magenta), 50 nM VASP-Halo:JF549 (yellow), 50 nM α-actinin-Halo (unlabeled), 0.5 μM 30% ATTO-488 labeled G-actin, and 2 μM profilin. Scale bar, 2 μm. **B)** Montage of bundle reconstitution assay featuring labeled α-actinin and unlabeled VASP. Assay was prepared with 30% ATTO 488-labeled F-actin (cyan), 25 nM zyxin-Halo:JF646 (magenta), 50 nM VASP-Halo:JF549 (unlabeled), 50 nM α-actinin-Halo (yellow), 0.5 μM 30% ATTO-488 labeled G-actin, and 2 μM profilin. Scale bar, 2 μm. **C)** Representative TIRF snapshots of bundle reconstitutions at time = 600 s where indicated components were omitted. Scale bar, 2 μm.

bioRxiv preprint doi: https://doi.org/10.1101/2024.05.17.594765; this version posted May 18, 2024. The copyright holder for this preprint (which was not certified by peer review) is the author/funder, who has granted bioRxiv a license to display the preprint in perpetuity. It is made available under aCC-BY 4.0 International license.

D) Montage of a zyxin bridge (magenta arrowhead) which forms within a contractile network and accumulates VASP (yellow arrowhead), then ruptures. Assay was prepared with 100 nM zyxin-Halo:JF646, 50 nM VASP-Halo:JF549, 100 nM α-actinin-Halo, 1 μM 30% ATTO-488 labeled G-actin, and 4 μM profilin. Scale bar, 2 μm. **E)** Montage (left) and kymograph (right) of a laser ablation-generated strain site that fails to repair and ruptures. Vertical dashed lines indicate site of ablation, performed at time = 0 s. Time labels correspond to both montage snapshots and kymograph. Scale bar, 2 μm. **F)** Quantification of strain site repair frequency in the absence of VASP or α-actinin in assays otherwise performed as in D. Number of strain sites in each category are indicated; N ≥ 3 biological replicates. Conditions were compared by Fisher's exact test. **G)** Normalized actin fluorescence intensity (left) and VASP intensity fold increase (right) over time at strain sites that either repaired (blue, n = 17) or did not repair (grey, n = 7) in the presence of zyxin^{WT}. Vertical dashed lines indicate time = 0 s, when laser ablation was performed. Horizontal dashed line indicates baseline VASP intensity = 1. N = 5 biological replicates. Data are presented as mean ± SEM.

bioRxiv preprint doi: https://doi.org/10.1101/2024.05.17.594765; this version posted May 18, 2024. The copyright holder for this preprint (which was not certified by peer review) is the author/funder, who has granted bioRxiv a license to display the preprint in perpetuity. It is made available under aCC-BY 4.0 International license.

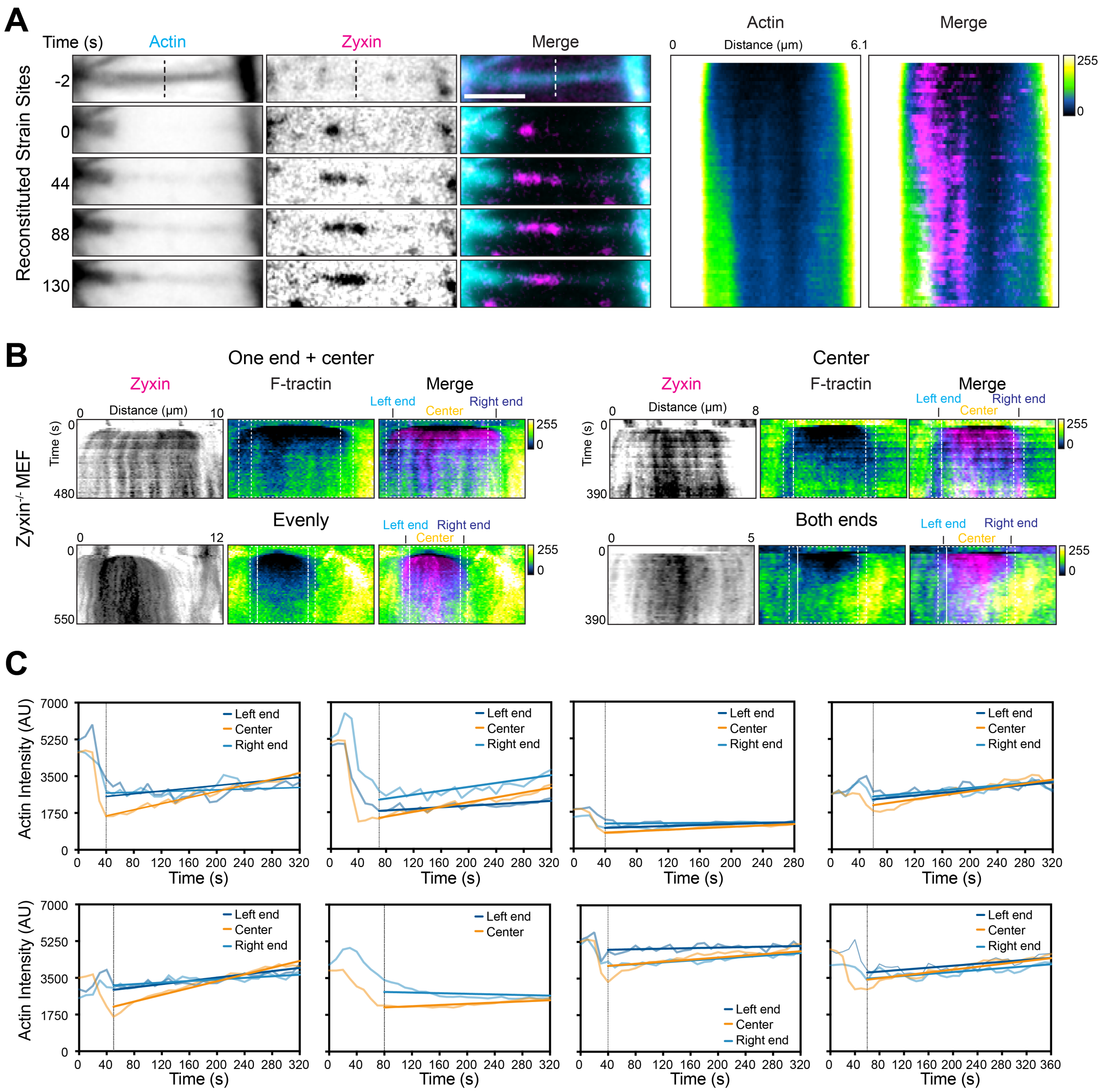

Figure S7. Additional analysis of SFSS actin repair paths. Related to Figure 6.
A) Montage (left) and kymograph (right) of a reconstituted strain site from a preparation lacking VASP, where actin recovery occurs at the strain site edges. Vertical dashed line indicates site of ablation, which was performed at time = 0 s. Time labels correspond to both montage snapshots and kymograph. Assay was performed with 100 nM zyxin-Halo:JF646, 100 nM α-actinin-Halo, 1 μM 30% ATTO-488 labeled G-actin, and 4 μM profilin. Scale bar, 2 μm. **B)** Kymographs of cellular SFSS representing each actin recovery path quantified in Figure 6D. Dashed boxes highlight quantified regions; each end was assigned as 10% of the overall strain site length. **C)** Actin recovery rate quantification for all cellular SFSS compared in Figure 6F, analyzed as in Figure 6E.

bioRxiv preprint doi: https://doi.org/10.1101/2024.05.17.594765; this version posted May 18, 2024. The copyright holder for this preprint (which was not certified by peer review) is the author/funder, who has granted bioRxiv a license to display the preprint in perpetuity. It is made available under aCC-BY 4.0 International license.

Table S1: Component concentrations in force reconstitution assays

Force Reconstitution Assays	Zyxin (nM)	VASP (nM)	α-actinin (nM)	Profilin (μM)	G-actin (μM)	ATP (mM)
Zyxin alone (Fig. 1)	25 / 250	0	0	0	0	0.5
Zyxin + VASP (Fig. 2)	25	50	0	0	0	0.5
Zyxin + α-actinin (Fig. 3)	25 / 250	0	50	0	0	1
Zyxin + VASP + Profilin-G-actin (Fig. 4)	25	50	0	2	0.5	2
Zyxin + VASP + α-actinin + Profilin-G-actin (Fig. 5,6)	25	50	50	2	0.5	2

VIDEO LEGENDS

Video S1. LIM protein bridges tether broken F-actin fragments. Related to Figure 1.
Representative movies of LIM protein F-actin-bound patches, bridges, and tails. Zyxin's LIM domains are sufficient to form bridges. FHL3, another mechanosensitive LIM protein, can also form bridges. Scale bar, 2 μm. Time is minutes : seconds.

Video S2. VASP dynamics on zyxin patches. Related to Figure 2.
Representative movies of VASP binding to zyxin patches. Scale bar, 2 μm. Time is minutes : seconds.

Video S3. α-actinin–zyxin binding and F-actin bundling dynamics. Related to Figure 3.
Representative movies of α-actinin binding to zyxin patches and clusters in the presence and absence of force (+/- ATP). Zyxin patches serve as a molecular guide for α-actinin-mediated zippering of translocating actin filaments into aligned bundles. Scale bar, 2 μm. Time is minutes : seconds.

Video S4. Dynamics of Zyxin, VASP, and α-actinin droplets. Related to Figure S4.
Representative movies of droplet fusion and fluorescence recovery after photobleaching (FRAP). Scale bar, 2 μm. Time is minutes : seconds.

Video S5. VASP-mediated F-actin nucleation and polymerization-coupled extrusion at zyxin patches in the presence of myosin forces. Related to Figure 4.
Representative movies of immobilized myosin V and myosin VI conditions. Scale bar, 2 μm. Time is minutes : seconds.

Video S6. Dynamics of reconstituted actin-myosin bundles and zyxin-mediated repair. Related to Figure 5.
Representative movies of reconstituted non-contractile and contractile bundles, including examples of spontaneous zyxin bridge formation, zyxin flashes and zyxin mediated repair, as well as catastrophic bundle rupture at a zyxin flash. Scale bar, 2 μm. Time is minutes : seconds.

Video S7. Dynamics of F-actin recovery paths at strain sites. Related to Figure 6.
Representative movies of F-actin recovery initiating in the cores of both reconstituted strain sites and SFSS of zyxin$^{-/-}$ MEFs expressing zyxin-mNeonGreen and F-tractin-mScarlet, followed by examples of all F-actin recovery paths observed in cells. Scale bar, 2 μm. Time is minutes : seconds.
